# Preferences of people in choosing a family physician in rural areas: a qualitative inquiry from Iran

**DOI:** 10.1017/S1463423622000317

**Published:** 2022-09-13

**Authors:** Mohammad Bazyar, Vahid Yazdi-Feyzabadi, Mona Bahmani, Jamil Sadeghifar, Khalil Momeni, Zahra Shaabani

**Affiliations:** 1 Department of Health Management and Economics, Faculty of Health, Ilam University of Medical Sciences, Ilam, Iran; 2 Health Services Management Research Center, Institute for Futures Studies in Health, Kerman University of Medical Sciences, Kerman, Iran; 3 Department of Health Management, Policy and Economics, Faculty of Management and Medical Information Sciences, Kerman University of Medical Sciences, Kerman, Iran; 4 Bachelor of Public Health, Health Research Deputy, Ilam University of Medical Sciences, Ilam, Iran; 5 Health and Environment Research Center, Ilam University of Medical Science, Ilam, Iran; 6 Social Determinants of Health Research Center, Institute for Futures Studies in Health, Kerman University of Medical Sciences, Kerman, Iran

**Keywords:** continuity of care, family physician, patient satisfaction, primary health care

## Abstract

**Background:**

Creating a stable and long-term relationship called ‘longitudinality’ between the population and general practitioners is crucial for the family physician program. The constant change of family physicians (FPs) can deteriorate longitudinality. This study aims to reveal what factors people usually consider when choosing a new FP or changing their current FP.

**Method:**

A qualitative study with a thematic analysis approach was carried out in Ilam province, Iran, in 2019. Purposeful sampling with a maximum variation strategy was followed to select the key informants. We did 34 interviews with following groups: patients (rural residents); FPs; and experts from Iran Health Insurance Organization, Ilam University of Medical Sciences, and Health Network Development Center. Data were analyzed using a thematic analysis to identify and contextualize the preferences of people in choosing a FP in rural areas. All the processes related to data coding and emerging themes were carried out using MAXQDA 2012 software.

**Results:**

The content of the interviews was categorized into 2 main themes, 6 sub-themes, and 39 codes. The first theme was ‘family physician characteristics’ including four sub-themes: general behaviors, social and physical characteristics, professional expertise, and pharmaceutical prescriptions. The second theme was ‘health center’ consisting of two sub-themes including location and physical features and properties of the health center.

**Conclusion:**

Some of the factors extracted from the interviews may have a different effect on the choice of people with different demographics. For instance, patients may have different ideas about the age, gender, years of medical practice, and finally, language and origin of the birthplace of FPs. Quantitative studies are needed to rank the factors identified in this study according to their significance for choosing FP and reveal patients’ preferences for each factor.

## Background

Since the mid-1980s, the Islamic Republic of Iran has enjoyed an extensive network of publicly funded primary health care (PHC) services in rural areas (Bazyar et al., 2016). A wide range of public health services is provided by a health network at the first level of the health system, which is managed by a general physician. These services include vaccinations, prenatal and postnatal care, growth monitoring, mother and child services, common infectious and chronic conditions management, mental health problems, and professional and environmental health services (Takian, Rashidian, and Kabir, 2011; Khatami *et al.*, 2020).

Unlike PHC, secondary health services were less accessible for the rural population due to financial and organizational obstacles, obligation to follow the referral system, and health insurance coverage with a weak benefits package. The fourth national development plan (2004–2009) passed a law to increase the public budget for the rural health insurance scheme to advance health equity and healthcare utilization for the rural population. The law emphasized on implementing family physician program (FPP) as the main mechanism to organize and provide healthcare services for the rural populations, and it was extended to all rural areas and cities with populations of up to 20 000 (Rashidian et al., 2013; Doshmangir *et al.*, 2017; Bayati *et al.*, 2020). In the fifth and sixth national development plans, FPP was also emphasized again to be extended to the whole country (Doshmangir et al., 2017). In 2011, the Ministry of Health and Medical Education (MoHME) cooperated with the Ministry of Cooperatives, Labor, and Social Welfare decided to expand FPP policy to urban settings with 20 000–50 000 population across the country. To extend the program to urban areas and all over the country, FPP has been implemented in two provinces of Fars, in the south of Iran with a population of around 4.4 million, and Mazandaran, in the North of Iran with an approximate population of 3 million, as a pilot program since 8th of July 2012 (Honarvar et al., 2015; Fardid *et al.*, 2019).

### Structure of district health network in Iran

**Figure 1.**
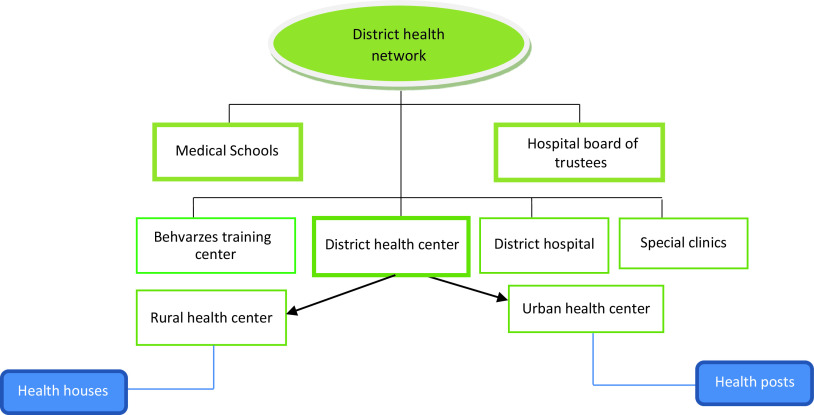
Structure of district heath network in Iran

The Iranian healthcare system is organized on three levels, including the national level (MoHME), provincial level (universities of medical sciences), and district level (health network executive units). Figure 1 illustrates Iran’s current district health network (DHN) structure. The main purpose of the structure of DHN is to bring public health services to where people live and make them accessible as much as possible. The most peripheral health facilities in rural and remote areas are health houses. They cover approximately 1500 population who live in the main and satellite villages. Two female and male community health workers (Behvarze) work in each health house. Behvarzes are usually from the same village where they work. They have basic education and provide PHC for the villagers. The Behvarzes provide public healthcare services, including annual census and registration of health information, health education, maternal and child care, family planning, nutritional care and improvement, school health, oral health, immunization, and environmental and occupational health. If patients need more professional health services, they are referred by the Behvarze to the upper level, rural health centers (RHCs), run by family physician (FP) and health technicians. The different health workers in RHCs, where the FPP is running, include FPs as the head of a health team, dentists, midwifery, public health, occupational health, environmental health technicians, and nurse and nurse assistant. In some RHCs that cover a large population and are far from other cities, other health workers such as psychologists, nutritionists, laboratory, and radiology technicians are also available. RHCs monitor and guide the health houses’ activities, provide outpatient care, and refer more complicated cases to the upper level, which is the district hospital. In 2020, there were 6973 FPs, 5669 midwiferies, 1899 dentists, 138 dentistry technicians, 806 nurses, 1503 laboratory technicians, 98 radiology technicians, and 4661 health technicians in the RHCs (Doshmangir et al., 2019; Khedmati *et al.*, 2019; Yazdi-Feyzabadi, Bazyar and Ghasemi, 2021).

According to the FPP instruction, a FP is a doctor responsible for covering the health services for a defined population within a specific catchment area in the first level of the health system. Holistic understanding of people and the context in which they live; comprehensive and integrated responses to provide a wide range of healthcare services for people; and continuity of care are the main distinct features of PHC. These features all depend on a stable, long-term, personal relationship, also called ‘longitudinality,’ between the population and the professionals who provide PHC services at the first level of health system. Different features may break this continuity, such as weak electronic health information system, paying for health services, changing the living place, and travel expenses (Grone and Garcia-Barbero, 2002; WHO, 2008).

Another overlooked factor that can deteriorate the continuity is a constant change of FPs, reducing the effectiveness of healthcare services. The population can change their FP if they are not satisfied with their current one. Studies from other countries also show that people see different PHC doctors if they are not satisfied with their last appointment. In FPP, where we look to create a long relationship between the population and FPs, a satisfactory choice and factors that may influence this choice are important (Wun et al., 2010). Many different pieces of research have studied the effects of FPP implementation and the facilitators and obstacles to establishing and developing FPP in Iran. However, few studies have investigated the factors which may influence people’s choice of FP (Khatami et al., 2020). The current relevant studies have mainly focused on choosing a doctor, not a FP, with distinct features mentioned above. Other studies showed that choosing a new doctor can be difficult and deserves particular attention (Wolinsky and Steiber, 1982).

A better understanding of the factors that influence people’s choice of healthcare providers would provide them with the resources to make better choices in this arena and create a better, long-lasting relationship between the population and the FP (Bornstein, Marcus and Cassidy, 2000). It is widely believed that people have difficulty finding out about doctors and therefore have little effective choice when they need a new general practitioner (Salisbury, 1989). In Iran, with its own social, cultural, and religious values, it is more important to know what may affect the physician–patient relationship. Finding the answers can be informative for policymakers working on the FP policy in other countries. It can help Iranian decision-makers make necessary modifications in medical education courses, specifically for family medicine, introduced in Iran in 2012. So, in this study, we aim to reveal what factors people usually take into account when choosing a new FP or changing their current FP.

## Methods

### Study design

This qualitative study with a thematic analysis approach was carried out in Ilam province, in 2019 aiming to explore the perceived factors that influence people’s choice of a new FP or changing their current FP. Thematic analysis is an insightful research approach that systematically identifies, organizes, and provides insight into patterns of meaning (themes) in a dataset. By focusing on the meanings across a dataset, thematic analysis allows the researcher to see and understand collective or shared meanings and experiences (Cooper et al., 2012). It extracts the components or important parts of the existing data related to the topic and the research question being studied and interprets the results by presenting data in themes that involve understanding, interpreting, and conceptualizing, and contextualizing the underlying meanings of the qualitative data (Braun and Clarke, 2014).

### Setting

As one of the 31 provinces of Iran, Ilam province is the least populated province, with 580 158 people according to the 2016 census. Composed of 10 counties, Ilam province is located in the western part of the country, sharing 425 km with the border of the country of Iraq. Ilam has a diverse linguistic composition. Kurdish, especially the Southern Kurdish dialects including Kalhori, Elami, Laki, and the Khezeli dialect spoken dominantly by 85.7% of the population, followed by Luri with 10.7%. Although Iran’s formal and national language is Persian, speaking Persian in Ilam province is not widespread. Laki and Arabic are also spoken by people, mainly in southern parts of Ilam province. Ilam province is mainly formed of Shi’a Muslims (about 4–8% of the population of Iran is Sunni Muslims) (Ghasemi, Momeni and Bahmani, 2013; Aliakbari, Gheitasi and Anonby, 2015)

#### Participants and interviews

Table 1 shows the number of current RHCs implementing the FPP in the health network in Ilam province. Currently (2021), there are 52 FP centers in rural areas in Ilam province. Some are single-FP centers; some of them have two FPs, and others have more than two FPs according to the size of the population within their catchment area.

**Table 1.** Number of family physician centers in health network in Ilam province, 2021

	Number of health center
Health centers with a single-family physician	27
Health centers with two family physicians	12
Health centers with three or more family physicians	13
Total	52

In line with the purpose of the study and catching the viewpoint of all relevant stakeholders knowing and having experience about what may matter to people when they choose a FP, purposeful sampling with maximum variations was followed. Following groups were included for interviews: rural residents, general physicians working in rural health facilities as FPs or those with similar experience in the past, and administrative staff from organizations with close relationships with FPPs, including Iran Health Insurance Organization, Ilam University of Medical Sciences, and Health Network Development Center. More details about the characteristics of the interviewees, including the age, gender, living place and language of each interviewee, and length of each interview, are presented in Table 2. Rural residents and FPs were interviewed in the RHCs. We chose patients (rural residents) attending RHCs for interviewing. As all patients referring to the health center could potentially give us information regarding the subject, we used convenience sampling and asked patients to participate in the study. Most of the rural residents interviewed for the study were women as, generally, they are the main visitors to primary health centers to get maternal and child care. We interviewed the FPs in health centers, but for four FPs in remote areas, we contacted them via telephone. To extract more information, we selected FPs with the following features: those who had worked as FPs for a long time, worked in different regions in Ilam province, and, most importantly, those who had the experience of working in the health centers with two or more FPs. Health centers with two or more FPs can provide better situation to increase the right of people to choose among physicians. This helps people to compare physicians with each other and understand the differences between them which in turn could help interviewees (rural residents and FPs) to reveal more details about the reasons why they may change their doctor, create a longer relationship with a doctor or prefer a doctor over another doctor. In general, 34 interviews were conducted. Data collection and interviews continued until saturation was reached, a point in which ‘no additional data are being found’ (Fusch and Ness, 2015).

**Table 2.** The number and main features of the interviewees

Setting	Organizations, Institutions, political groups, and individuals	No.	Length of interview	Gender and place	Language spoken	Age
Ilam District Health Network	General doctors working as rural family physician	11	Minimum 25 min, maximum 1 h, average 40 min	6 female, 5 male (9 Ilamian and 2 non-Ilamian)	8 Kurdish, 3 Persian	Range of 7 of FPs were between 26 and35, other physicians were 47, 53, 55 and 58 years old
	Patients (rural residents)	19	Average 10 min	17 female, 2 male (all Ilamian)	Kurdish	They were mainly young, some of them were adult
	Health Network Officials	1	25 min	Female (Ilamian)	Kurdish	46 years old
	Community Health Workers (Behvarze)	1	20 min	Female (Ilamian)	Kurdish	44 years old
Ilam Province Health Insurance Organization	Department of Supervision	2	20 min	Female (Ilamian)	Kurdish	38 and 46 years old
Total		34				

### Interview guide

Three in-depth unstructured interviews were conducted first with FPs to collect the data. These initial interviews resulted in a semi-structured interview guide to conduct the rest of the interviews. At the start of each interview, by explaining the purpose of the study and ensuring the confidentiality of the content of the interviews and anonymity, interviews were taped with two sound recorders, and immediately the recorded interviews were transcribed verbatim by one of the authors, who is fluent in Kurdish accent/language.

### Data analysis

One author (MB) initially indexed the transcribed interviews. A thematic analysis was used to analyze the data based on the methodology highlighted by a six-step framework proposed by Braun and Clarke including familiarizing with data, generating initial coding, searching for themes, reviewing of themes, defining and naming of themes, and reporting the findings (Braun and Clarke, 2006). As the analysis was grounded in the data, an inductive approach highlighted patterns and similarities. Any identifying characteristics were removed from the quotes, and identity information was replaced with general characteristics such as their position, gender, and age. The next stage of analysis involved coding the generated data into initial themes. The themes were generated from the patterns within the codes and were included when they linked to the research aims, were frequently mentioned, and appeared important to the participants. Later on, a comparison of the initial themes was conducted and finally grouped into non-overlapping broader themes that were defined and specifically organized to address the research question of criteria to choose and select a physician. The themes have been illustrated with paraphrased extracts from the quotes. Direct quotations have been used to match the data with the themes that emerged. All the processes related to data coding and emerging themes were carried out using MAXQDA 2012 software (VERBI Software. *MAXQDA12*. Berlin: VERBI Software, 2012).

#### Trustworthiness

To ensure the quality of results, we employed four trustworthiness criteria suggested by Lincoln and Guba (1986). Credibility was met with a prolonged engagement whereby the principal investigator (MB) continuously worked for nearly 4 months with the qualitative data. Furthermore, member-checking validation was used by delivering some transcribed interviews to the respective participants. They then asked them to ensure a good correspondence between their findings and the participants’ perspectives. To improve credibility, particularly for this study, we focused on the contrary and opposite cases to provide a comprehensive picture derived from different perspectives. A detailed description of the DHN in Ilam and applying a maximum variation sampling technique were utilized to enhance the transferability and reflexivity of the findings. The dependability of the research was assured by an auditing approach in which the VYF, accompanied by an external auditor, engaged in critical comments in the coding process and analyzing of transcribed interviews as well as cross-checked the data that we collected. To increase confirmability, we employed data source triangulation approach (Carter *et al.*, 2014), including interviews with patients, FPs with different backgrounds, and experts from other relevant organizations familiar with FP program. All methods were carried out under relevant guidelines and regulations.

### Findings

The content of the interviews was categorized into 2 main themes, 6 sub-themes, and 39 codes. Table 3 shows the main findings briefly.

**Table 3.** Themes, sub-themes, and codes related to the factors which may influence decision of people in Ilam, Iran, choosing their family physician

Themes	Sub-themes	Codes
Family physician characteristics	General behaviors	Kindness
		Being patient to listen to patients’ questions and concerns
		Treat all patients equally
		Being humble and modest
		Being chaste
		Making a friendly communication with patients
		Caring about non-medical problems of patients and their families
		Considering Islamic and religious values
	Social and physical characteristics	Being native or non-native (being from Ilam province)
		Having previous acquaintance with physician
		Language
		Age and gender
		Ethnicity
		Wearing medical uniforms
		Neat and tidy appearance
		Having authority to bring about development in the region
	Professional expertise	Precise and accurate diagnosis
		Putting enough time for medical examination
		Availability and attendance of physician in health facility
		Getting the health services in the shortest time possible
		Caring about and visiting people living in remote catchment areas
		Following up the health status of patients especially those with chronic conditions
		Doing additional things (such as wart and mole removal) in addition to the medical practices
		Preferring a physician less strict to refer patients to specialists
		Taking into consideration the enmities between people while treating them
		Having a substitute physician when the physician is not available
		Providing advices about healthy lifestyle alongside medical prescriptions
	Pharmaceutical prescriptions	Precise and deep explanation regarding medicines prescribed for the patients and how they should be taken
		Getting medicine free of charge
		Consider the preference of patients regarding the kind of a medicine they want such as tablet, liquid, capsule, and injection
		Number of medicines prescribed in each prescription
		Consider the exact and specific medicines (even the color of tablet) that patients request for
		Prescribing medicines the patients are looking for not something which is needed based on physician’ knowledge
Health center	Location	Proximity of people’s place of residence to health center (distance between them)
		Being near to the shopping centers and other facilities like amusement park
	Physical features and properties	Preferring a health center with multiple family physicians rather than a single-family physician
		Having an active pharmacy inside or near to the health center
		The physical appearance of the health center
		The quality of health equipment in the health center

We explain the codes in detail as follows.

#### 1: General behaviors of FP

##### Listen to the patients patiently

According to the interviews, people want to be heard patiently. They may talk about their life problems, sometimes, they do not need any specific medicine, but they come just to talk with the doctor about their concerns. So FPs should be patient to listen to their concerns. It is all that they need in some cases.

*‘Family physician should be kind with patients, talk to them and listen to their problems patiently. Most of the patients come just to confide their life problems to me’ (A mature female family physician)*

##### Considering religious values

The main religions in Iran are Shi’a and Sunni. Ilamians are Shi’a. Although interviewees did not mention religion as an important factor, however, some interviewees emphasized on considering general Islamic values like having Hijab (Islamic cover for women), especially in rural areas and small cities where most people know each other and are meticulous and strict about considering Islamic values.

*‘Here is a rural area, people have their own strict religious beliefs, and we as doctors must respect at least the usual religious values as much as possible. We had once a female colleague whose Hijab was not proper; it raised many challenges’ (A young male family physician)*

Kindness and treating people from different socio-economic backgrounds kindly and equally, showing respect and good manners, being humble and modest, and being chaste were among other general features that patients stated a lot. Some FPs also mentioned that people consider physicians as knowledgeable persons. They usually consult them about other non-medical problems. This point can greatly affect people’s satisfaction and create a strong relationship between them.

#### 2: Social and physical characteristics

##### Being native or non-native

Being from Ilam originally or coming from other provinces to work in Ilam as a FP is a factor that may influence people’s behavior and trust toward physicians. According to the interviews, there is no fixed answer for it; Ilamian physicians can be both good and bad, according to the time and context. Iranian, in general, and Ilamian, in particular, shows respect and hospitality and are so kind toward strangers and people from other region. In general, being native is better as it causes fewer language and cultural problems, but people also respect physicians from other provinces.

*‘In my opinion, being non-native is an advantage for a doctor who works in Ilam province. People have a special trust in a doctor who is non-native, it is completely obvious’ (A mature non-Ilamian male family physician)*

*‘We prefer physicians from other cities; we say they are from other cities, they know more, they are more experienced’ (A 36-year-old female patient)*

### Ethnicity

Ilam province is composed of several ethnic groups. Most interviewees mentioned ethnicity as a factor that can influence the relationship between physicians and patients. Being from the same ethnicity was mentioned as a factor that can be either good or bad. Most interviewees said that being from the same ethnicity is preferred as it can affect other factors like language and accent. They may trust each other better and help them create a friendly atmosphere sooner.

*‘I am a physician from Shoohan ethnicity, and if someone who comes to me is from Shoohan, he/she will be happy and says the doctor is from my ethnicity, they feel better. Or if I have a patient from Malekshahi ethnicity and he knows I am Shoohanian, they say we are like brothers and sisters. If physician and patient are from the same ethnic group, they usually trust each other more quickly, but it is not always the case’ (An old Ilamian male family physician)*

Some interviewees had different ideas. They believed being from different ethnicity is better in general. If the relationship between ethnicities is stained, they may generalize this to the relationship between physicians and patients.

*‘If you made a medical error or your prescription resulted in an adverse effect, they would say ‘noticed? He did it deliberately as he feels enmity towards us. I’ve witnessed this in my work’ (An old Ilamian male family physician)*

Another factor originating from being native is having previous acquaintance with a physician. Knowing the physician from the past can be likened to a two-blade sword. This previous acquaintance can be both positive and negative depending on the physicians’ previous history and even the reputation of their families.

*‘They may say (in a ridiculous manner) that miserable so-and-so is going to be our doctor, or on the contrary, they may say thankfully he/she is originally from our region and now is working for us as a physician, and we can share with him/her our problems’ (An old Ilamian male family physician)*

### Language

The main language spoken in Ilam is Kurdish. According to the interviews, as most people here in Ilam speak Kurdish and their mother tongue is Kurdish, it is easier for them to speak and communicate in Kurdish, especially for adult and older adults and those who live in deprived areas. Some interviewees mentioned that it is advisable to use different languages for different people, for instance, Kurdish for elders and Farsi for the younger generation.

*‘At the beginning of my work, I thought that if I speak Farsi, patients will listen to me carefully, and if I speak Kurdish, they think I am someone uneducated like themselves, but that’s not the case. There are many concepts in the Ilamian language that you can’t find an appropriate equivalent for them in Farsi. I am from Ilam, and when patients speak about their medical problems in Kurdish, I know exactly what they mean. This issue is true, especially for elders; they can communicate easier and better when facing a Kurd physician. Many times, it has happened to me that what patients told me in Kurdish was completely different from what they tried to communicate in Farsi’ (A young female physician).*

*‘Kurdish language physician is better here; here is village, most of the people are low-educated (Speaking Farsi is not easy for them)’ (A 31-year-old female patient)*

### Gender and age

In Iran, women usually go to healthcare facilities for mother and child health services. Due to Iran’s cultural, social, and religious values, the general belief may be that women prefer female FPs. Interviewees raised different ideas. Some interviewees said there is no difference, and patients do not care about the doctor’s gender. Other patients mentioned that female doctors are preferred for gynecological diseases. Some believed that, in general, patients trust male physicians, especially old physicians, more than young female ones in terms of accuracy of prescription and treatment.

*‘Because of strong patriarchy here, patients prefer male physicians, particularly if the physician is senior. A senior male physician is much preferred than a young female physician’ (a female family physician). ‘The fact is that people here trust a male doctor more’ (A young Ilamian male family physician)*

*‘If I have a medical problem which I cannot share it with a male physician I prefer a female one, otherwise and for ordinary problems like cold, I go to a male physician cause I think they are more (medically) reliable’ (An adult woman patient)*

*‘Between a young and old physician, I would choose an older physician, as they are experienced’ (A 20-year-old female patient)*

### Having the authority to bring about development in the region

One point inferred from one of the interviews was having a close and intimate relationship with top health officials and authorities in the province to bring new financial resources or medical facilities into deprived areas. Health facilities in different parts of the province are not equally equipped, and one influencing factor is health managers’ power and political lobbies. As the FP is the head of the health center in each RHC, they can bring changes to the region. It may not be an important criterion for people, or they may not be aware of it when choosing a general practitioner. However, it can influence the public’s satisfaction in the region.

*‘When I began my work as a FP, the physical condition of the health center was very bad. There were lots of spider nets around the health center. There were not even enough garbage cans in the health center. I used my relations with the top health managers in the region to repair the center. People know these changes happened because of me. Once I wanted to leave this center, people came to me and didn’t allow me to leave’ (A young Ilamian male family physician).*

The interviewees also mentioned wearing medical uniforms and having a neat appearance as influencing factors. In general, patients stated that they prefer a physician wearing a medical uniform. However, they cared more about other criteria, such as professional expertise and medical behaviors, explained below.

#### 3: Professional expertise

##### Precise and accurate diagnosis

One of the main criteria mentioned by most of the interviewees was the physician’s knowledge and ability to diagnose the patient’s problem accurately. As interviewees said, this criterion was even more important than other criteria such as kindness, gender, ethnicity, religion, and language. As some interviewees mentioned, when a general physician cannot solve the problem, it is important to refer the patient to an appropriate specialist. Patients do not like to wander in the health system. Another related issue that rose was taking into account the socio-economic condition of the patients. When prescribing medicines, the physician should ask patients about their affordability.

*‘The first thing that I realized (people care about) was physicians’ medical knowledge to deal with a wide range of health problems. The family physician should be alert and can answer almost all questions raised by patients’ (A young female family physician)*

*‘I come to FPs for my health problem, so it is important how they cure me, their precise diagnosis is much important for me, rather than their behavior’ (A 20-year-old female patient)*

##### Putting enough time for medical examination

Putting enough time to examine the patient carefully and listening to what patients say is so important for patients. Most complain about not being listened to when they go to a doctor.

*‘My husband and I have blood pressure and taking pills, but we don’t go to our health center to visit the family physician because he/she does not put enough time for us’ (a mature female patient)*

##### Availability and attendance of physicians in the health facilities

According to the interviews, people care a lot about the doctor’s presence when they go to the health center. Usually, in the main village where the health center is located, the first priority for people is that the FP should come on time and go on time, and people want to get their services without delay.

*‘Family physician should be at work on time’ (A 20-year-old female patient) ‘the doctor and midwifery should be present at the center all the time’ (A 31-year-old female patient)*

*‘The regular attendance and punctuality of physicians is quite important, many times I came to work at 8 am, and I saw people waiting for me’ (A young female family physician)*

##### Getting the health services in the shortest time possible

People want to get the healthcare services they need very soon. They do not like to wait. They expect the FP to visit all patients in a short period.

*‘Here in the region, people are impatient generally. They always nag about the waiting time in the health center and have complained to me about why it takes so long to visit all patients and why I put too much time on visiting each patient. Whenever I am off for a day, and another doctor is here to visit the patients, they become happy and admired him or her for quickly visiting all patients!’ (A mature non-Ilamian male family physician)*

##### Caring about people living in remote places within catchment areas

According to the principles of the DHN, within a catchment area, health centers usually are located in the main and populous village, and other small or remote areas should be covered by FPs and their health team regularly. So covering the remote areas on a regular schedule is important, affecting public satisfaction.

*‘We don’t have a family physician here. The visiting plan of the family physician and midwifery is on Sunday and Thursday, but usually either midwifery or the doctor doesn’t come’ (A 31-year-old female patient)*

*‘Going to hard-to-reach areas is very important for people’ (A young male family physician)*

##### Following up on the health status of patients

A FP is responsible for people’s health in their catchment area. This point is more important for those who struggle with chronic health conditions. According to the interviewees, caring about and following up on the health status of people with chronic condition can remarkably increase people’s satisfaction.

*‘When the family physicians refer you to other health technicians to complete your treatment, they should follow up on your treatment and care about you to see if other health technicians do their job right’ (a young female patient)*

*‘For patients who need following up and constant care, I ask health workers in remote areas to look after them. This following up is so important; this is so satisfactory for people’ (A young male family physician)*

#### 4: Pharmaceutical prescriptions

##### Physician’s principles regarding medicine prescription

Regarding prescribing medicines by the FPs, different issues are raised that affect the patients’ judgment. Patients want physicians to explain reasons for prescriptions and explain how to take them. Patients have different expectations regarding the medicines prescribed by the FP. Some patients expect FP to prescribe a lot of medicines for any medical problem; some of them only ask for a specific kind of tablets, for example, they may take tablets with a specific color (they do not accept the same tablet with a different color); some patients expect to get all kinds of medicines freely, especially those who live in remote areas; or some of them may prefer a different kind of medicine such as a tablet, liquid, capsule, and injection. Interviewees mentioned that some patients force them to prescribe whatever they are looking for, whether it is appropriate for them or not; this may be a medicine that has been effective for someone else or another disease.

*‘In this center, it was common to get medicine, multivitamin, iron, and magnesium tablets for free previously, but now it is not free and available anymore, just pregnant women can get them. There is lack of medicine here’ (A 31-year-old female patient)*

*‘I have seen a lot that my colleague, pharmacist, explained for the patient how to use medicines, but they came to me after that, looking for more explanation’ (A young female family physician)*

*‘This health center has two family physicians; when I don’t prescribe injections for the patients, patients get a new admission to get whatever they like by the other physician’ (A mature female family physician)*

#### 5: Location of health facility

##### The distance between the living place and the health facility

Physical accessibility affects the utilization of healthcare services by people. Interviewees said they prefer a closer health center to get their health services.

*‘My home is just in the alley next to the health center. I did my domestic chores and came here (the health center) just by my casual clothes. If the center was far away, I had to put on my make-up or change clothes (dress up)’ (A 31-year-old female patient)*

*‘I’ve been recently providing health services in two health centres, the old one which is under repairmen and renovation now and this center that I work in. Those patients who trust me come to me no matter how far the health center is. Those who were so-so did not come to me again as this new health center is farther; those who live in the neighborhood come more; for example, the opposite neighbor comes every day to control her blood sugar. Those who are farther now complain asking me to come to the previous location’ (An old male family physician)*

#### 6: Physical features and properties

##### Preferring a health facility with several FPs rather than a single FP

Interviewees mentioned that people prefer health centers with several FPs in crowded villages as they have several options to select among them. A health center with several FPs means that there would also be at least one physician available in the health center most of the time to visit people.

*‘People prefer a health facility with multiple family physicians rather than a single-family physician, because they would have more doctors to choose or when their family physician is not available, other doctors can cover their absence’ (A staff from the Ilam province branch of the Iran Health Insurance Organization)*

##### Having an active pharmacy inside or near the health facility

Findings revealed that having an active pharmacy within or near to the health center to provide the medicines that people need is so satisfactory for

*‘There always should be a pharmacy here, not just only the days which family physician comes here to visit us’ (A young female patient)*

*‘People prefer a health center where a pharmacy is available or nearby’ (A staff from the Ilam province branch of the Iran Health Insurance Organization)*

The interviewees also mentioned that the physical environment of the health facility and the quality of health equipment such as computers and beds, and the cleanliness of the FPs’ workplace influence the judgment and choice of FP by rural people.

## Discussion

In this study which was done in a small province in the west of Iran with its specific social and cultural features, different criteria were mentioned by the interviewees that can influence the people’s choice of FP. What matters in people’s decision to select a physician is always a challenging discussion among health policymakers. There are numerous and various factors among the general population to suit their needs for selecting a physician. The current study showed that people prefer a FP with the following features: precise and accurate diagnoses, putting enough time for medical examination, and availability and attendance of the FPs in the health centers. According to these findings, to increase the satisfaction of people with the FP program, we suggest policymakers implement training and orientation programs for the FPs to put enough time for each patient and also be present early in the morning and more regularly at the workplace. In a similar study in the south of Tehran, Iran, the authors found that the most important factors in selecting FP were as follows: having a specialist degree in family medicine, performing accurate examinations with receiving a detailed medical history, and spending enough time to visit patients, respectively (Khatami et al., 2020). In a study by Hoerger & Howard (1995), it was found that expertise was the priority for women to choose a doctor for prenatal care (Hoerger and Howard, 1995). Another study in the USA concluded that patient satisfaction ratings for quality of care received, access, interpersonal skills, medical education and training, and board certiﬁcation are the first to fifth most essential factors in selecting a primary care physician by patients (Razzouk, Seitz and Webb, 2004).

Something worth mentioning is that although the findings indicate that patients want FP to put enough time into visiting them, they complain about the waiting time in the health centers and want to be visited as soon as possible. To improve the quality of PHC in FP program in rural areas in Iran, it is necessary to implement cultural programs in rural areas for people to know how important it is to put enough time for examining each patient, even though it increases the waiting time. A recent large-scale survey of American patients’ satisfaction revealed that the most frequent complaints of patients to get healthcare services were regarding long waiting times (Bornstein, Marcus and Cassidy, 2000).

The proximity of people’s place of residence to the health center (distance between them) was one of the factors that can influence people’s choice of FP. These findings are in line with findings from previous studies. People choose their doctors initially on convenience or recommendation by their friends or relatives (Wolinsky and Steiber, 1982; Salisbury, 1989; Wun *et al.*, 2010). Proximity and convenience are the rule for a part of the population. They like to go to any doctor nearby, either close to home or workplace, and they may focus on it in their initial choice, especially in a context in which people are free to go to every doctor they like. This study also emphasized that proximity is important for PHC services, and those near the health centers go to get their health services more regularly. So it is important to remove the geographical barriers as much as possible and visit people in remote areas more frequently.

However, findings from other studies show that in the long term and to create a strong doctor–patient relationship, people care more about the clinical proficiency of FP, reducing the importance of proximity. They would go back to the same doctor if they consider the doctor proficient clinically regardless of their proximity (Bornstein, Marcus and Cassidy, 2000; Thom, Hall and Pawlson, 2004). Interviews showed that people in Ilam province prefer a health center with several physicians rather than a center with only one physician. Currently, in health centers with several FPs, people can go to any of them when visiting the center. So they can go to other physicians in the center in the same day or the next time if they are not satisfied with the services they get. We suggest that in more populous areas, creating a multi-physician health centers is more advisable and can increase the availability of FPs for people. But the problem is that at the current situation in the health centers with several FPs, each time people can go to any of the FPs they like and population within the catchment area of health center is not divided between FPs. It means that no specific part of population has been assigned to each of the FPs. This can jeopardize the creation of long relationship between people and FP which is necessary for enhancing health of population and curing people in the context they live and work (Yazdi-Feyzabadi, Delavari and Ghasemi, 2018).

Our study also indicates that Ilamians respect non-Ilamian doctors who come to serve or work in Ilam province much more than Ilamian doctors. Even one of the interviewees from other provinces emphasized that people in Ilam highly hospitalize, respect, and trust non-Ilamian doctors, and this behavior is noticeable. Another interesting finding from our study is that interviewees, even female FPs, generally mentioned that patients trust male physicians, especially old ones, more than female physicians regarding medical and professional prescriptions. The national and provincial policymakers should consider this general mistrust that exists among people regarding the medical proficiency of the young female FPs, and corrective measures should be implemented to eliminate or alleviate this mistrust.

The physician’s ethnicity was another factor related to the physician’s social and physical characteristics, which the interviewees mentioned. Our study supports the idea of preference for a physician’s ethnicity to make communication effective considering the same language and accent. Ethnicity can influence the quality of the relationship between physicians and patients. In general, people may trust a physician of the same ethnicity easier and can find more commonalities, leading to a friendlier relationship. However, if the FP and patients are from different ethnicities or from different sub-groups of ethnicity within the region and there is enmity between them, this may influence the quality of the physician–patient relationship. One of the FPs said that FPs should consider the enmity between patients from different groups of ethnicities while treating them. For instance, in visiting remote areas with no health center, the FP should visit people in an impartial place such as a school or mosque instead of treating people in a place belonging to one of the ethnicities. A study in California, USA, aimed to investigate racial and ethnic differences in patients’ selection of surgeons and hospitals and concluded that minority groups including black and Hispanic women were less actively involved in physician and hospital selection. Therefore, their choice relied more on physician referral and health plans than on the provider’s reputation (Freedman et al., 2015). A study conducted by Abghari et al. (2014) in the USA showed that most patients had no preference for the race of their surgeon. However, those who preferred the race of the surgeon tended to prefer surgeons of their ethnicity (Abghari et al., 2014).

One interesting finding is that patients value a physician’s appearance, such as wearing medical uniforms and a neat appearance. This result further supports the idea obtained by Puhl et al. (2013) study indicating that the excess weight of providers may negatively affect patients’ perceptions of their credibility, level of trust, and inclination to follow medical advice (Puhl et al., 2013).

### Study limitations

In this study, we aimed to reveal more factors that may influence people’s choice of a FP, but it is not clear how different people with different demographic characteristics may value different criteria when choosing a FP. Quantitative studies are needed to quantify the importance of different criteria for people with different demographic characteristics.

## Conclusion

To improve the FPP in rural areas and for better development in urban areas in Iran, implementing corrective measurements is necessary to enhance the relationship between people and FPs. According to the study’s findings, it is advisable to consider the following recommendations. It is better to create health centers with several FPs in populous areas instead of creating several separate single-FP centers. The FPs should be trained about the necessity of putting enough time for each patient and being present at the work early in the morning. People expect to be visited as soon as possible, and they should be trained to be more patient and allow FP to put enough time for each patient. Policymakers should work to change the mistrust which generally exists in public about the medical proficiency of young female FPs. It should be mentioned that some of the factors extracted from the interviews may have different effects on the choice of different people with different demographics. For instance, different patients may have different ideas about the age, gender, years of medical practice, language, and origin of the birthplace of doctors. These items have no fixed negative or positive effect on people’s choice of FPs. Older patients may prefer doctors with the Kurdish language, while younger patients may have no problem with the Farsi language or even believe that those who speak Farsi are more knowledgeable than those who speak Kurdish. In this study, we tried to reveal the main factors influencing people’s choosing FP. Quantitative studies are needed to rank the factors identified in this study according to their significance for choosing FP and reveal patients’ preferences for each factor.
